# The intimate relationship between human cytomegalovirus and the dendritic cell lineage

**DOI:** 10.3389/fmicb.2014.00389

**Published:** 2014-08-07

**Authors:** John Sinclair, Matthew Reeves

**Affiliations:** ^1^Department of Medicine, University of Cambridge – Addenbrooke’s HospitalCambridge, UK; ^2^Institute of Immunity and Transplantation, University College London – Royal Free HospitalHampstead, London, UK

**Keywords:** latency, reactivation, dendritic cells, cytomegalovirus, inflammation

## Abstract

Primary infection of healthy individuals with human cytomegalovirus (HCMV) is normally asymptomatic but results in the establishment of a lifelong infection of the host. One important cellular reservoir of HCMV latency is the CD34+ haematopoietic progenitor cells resident in the bone marrow. Viral gene expression is highly restricted in these cells with an absence of viral progeny production. However, cellular differentiation into mature myeloid cells is concomitant with the induction of a full lytic transcription program, DNA replication and, ultimately, the production of infectious viral progeny. Such reactivation of HCMV is a major cause of morbidity and mortality in a number of immune-suppressed patient populations. Our current understanding of HCMV carriage and reactivation is that cellular differentiation of the CD34+ progenitor cells through the myeloid lineage, resulting in terminal differentiation to either a macrophage or dendritic cell (DC) phenotype, is crucial for the reactivation event. In this mini-review, we focus on the interaction of HCMV with DCs, with a particular emphasis on their role in reactivation, and discuss how the critical regulation of viral major immediate-early gene expression appears to be delicately entwined with the activation of cellular pathways in differentiating DCs. Furthermore, we also explore the possible immune consequences associated with reactivation in a professional antigen presenting cell and potential countermeasures HCMV employs to abrogate these.

## INTRODUCTION

Human cytomegalovirus (HCMV) remains a major cause of pathology in a number of patient populations. Pathogenesis correlates with an impaired immune response such that disease is particularly associated with congenital infection *in utero* or during infection and reactivation in immune-suppressed transplant patients as well as in immune-compromised, late-stage AIDS patients (Pass et al., 1983; Revello and Gerna, 2004; Legendre and Pascual, 2008). However, while an absence of a cellular immune response is a key factor, it has also been suggested that HCMV may also represent a clinical problem to critically ill non-immune-compromised individuals. For instance, in intensive care units, HCMV can present as a secondary complication – with the reactivation of latent HCMV representing the major source of infection (Papazian et al., 1996; Limaye et al., 2008; Kalil and Florescu, 2009). Pertinently, despite a number of strategies to control HCMV infection, the virus remains a major health burden.

The ability of HCMV to establish a latent infection likely contributes to the sero-prevalence within the healthy population. Furthermore, much of the well-documented, virus-associated pathogenesis in clinical settings results from reactivation of latent virus reservoirs. As such, understanding the underlying principles that govern HCMV latency and reactivation is important for deciphering the pathobiology of HCMV. In this review, we explore the intimate relationship between HCMV and myeloid dendritic cells (DCs). DCs, key antigen presenting cells (APCs), are both sites of reactivation from latency as well as primary lytic infection and thus have a central role in the lifecycle of HCMV. Indeed, the abundance of immune evasion molecules expressed by HCMV is testament of a close relationship with a pivotal cell type that orchestrates innate and adaptive immune response following infection.

## HCMV LATENCY AND THE MYELOID LINEAGE

**FIGURE 1:**
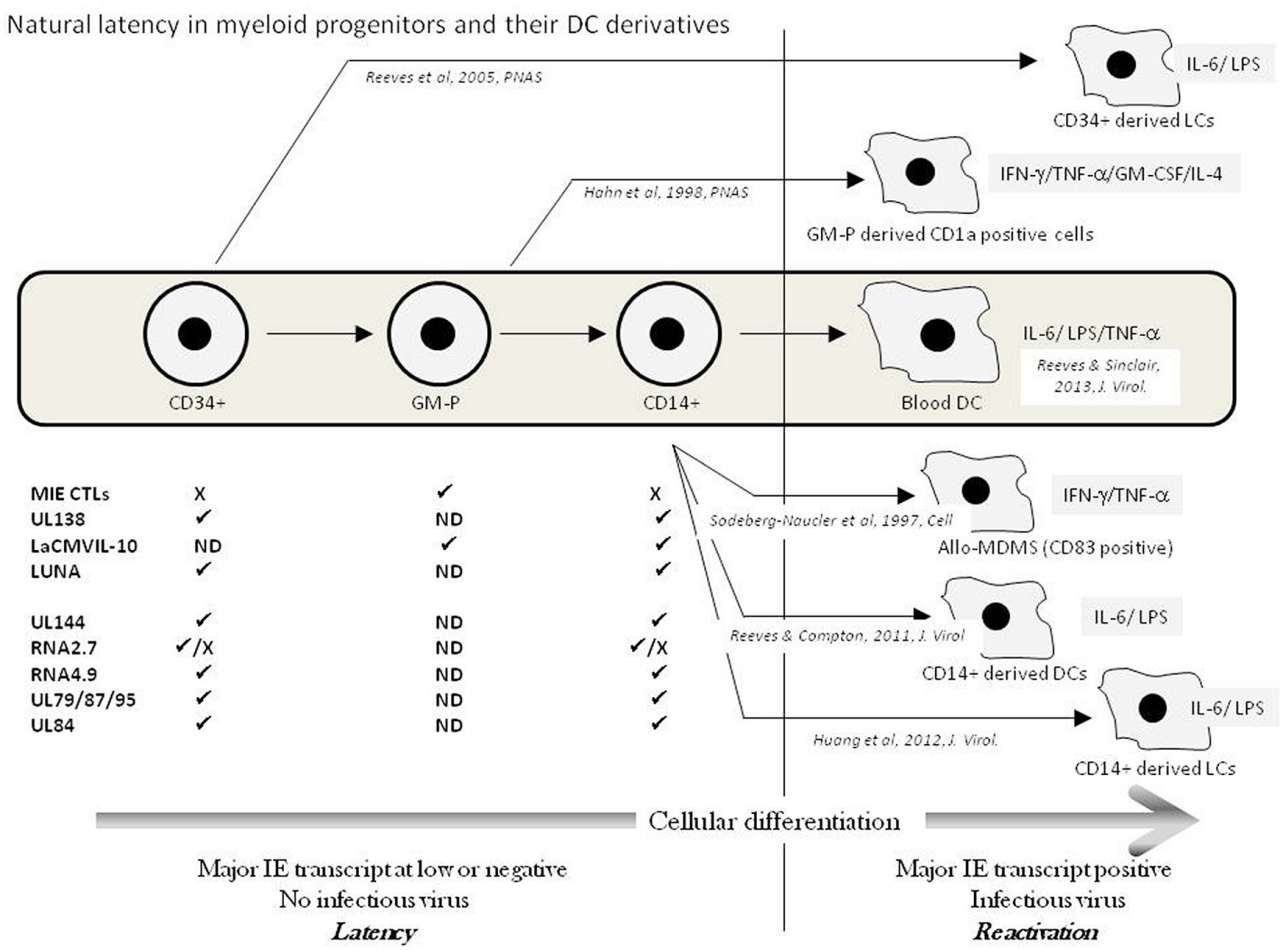
**Natural latency in myeloid progenitors and their DC derivatives.** The natural differentiation of HCMV from bone marrow progenitors (CD34+) via granulocyte-macrophage progenitors (GMPs) through to circulating CD14+ monocytes and blood DCs in the peripheral blood is illustrated (Grey box). Latency is maintained in pre-DC populations exemplified by a lack of major IE transcription and no infectious virus production. A number of transcripts have been detected in these different cellular populations (✓ = detected; X = failure to detect; ND = No Data) and have been suggested to represent putative latent transcripts. Upper transcripts CLTs to LUNA have been verified in multiple laboratories whereas lower transcripts await independent verification. The *in vitro* differentiation of myeloid progenitors to DC cell types that support HCMV reactivation is shown with the key cytokines implicated in reactivation illustrated from these studies of naturally latent myeloid cells.

It is now well established that HCMV can persist latently in haematopoietic progenitor cells resident in the bone marrow (Mendelson et al., 1996; Sindre et al., 1996; Zhuravskaya et al., 1997; Hahn et al., 1998). Despite the potential ability of these progenitor cells to give rise to both myeloid and lymphoid lineages, the detection of HCMV DNA in peripheral blood of naturally latent carriers is restricted to the myeloid lineage (Bevan et al., 1991; Taylor-Wiedeman et al., 1991, 1993). In such healthy individuals, this carriage of virus DNA in peripheral blood monocytes, but not T or B cells, is independent of infectious virus production (Taylor-Wiedeman et al., 1991, 1994). These observations are consistent with a lack of detectable infectious virus in the blood of healthy donors as well as early clinical observations which showed that HCMV transmission in blood transfusions was significantly diminished by prior leukocyte depletion (Gilbert et al., 1989). However, the reactivation of HCMV, *in vitro*, can be triggered from these early myeloid cells (CD34+ and CD14+ cells) by differentiation (Reeves and Sinclair, 2008). Some early studies showed that treatment of monocytes with mixtures of cytokines that promote macrophage differentiation could trigger the reactivation of immediate-early gene expression (Taylor-Wiedeman et al., 1994) and, in some instances, infectious virus (Soderberg-Naucler et al., 1997). Subsequent studies have now established that HCMV reactivation occurs in myeloid progenitors differentiated to DCs by specific cytokine stimulation (Reeves et al., 2005b; Reeves and Compton, 2011). Indeed, the first report detailing the reactivation of infectious virus from peripheral blood compartment cells of healthy seropositives required the allogeneic stimulation of monocytes (Soderberg-Naucler et al., 1997). The “cytokine storm,” associated with the allogeneic T-cell reaction, likely triggered virus reactivation and suggested a contribution of pro-inflammatory cytokines which, however, remained undefined (Soderberg-Naucler et al., 1997, 2001). Nevertheless, an interesting aspect of these studies was the phenotype of the differentiated monocyte: The cells reactivating HCMV co-expressed both macrophage (CD68) and, interestingly, DC markers (CD83). Parallel studies from the Mocarski laboratory, using a Granulocyte/macrophage – progenitor (GM-P) model, showed that reactivation of HCMV was concomitant with DC lineage commitment (based on CD1a expression) suggesting that DCs, as well as macrophages, were important sites of HCMV reactivation *in vivo* (Hahn et al., 1998). Subsequently, our own work has now shown that differentiation, *ex vivo*, of both CD34+ and CD14+ cells to specific DC phenotypes is important for reactivation (Reeves et al., 2005b; Reeves and Compton, 2011; Huang et al., 2012). Importantly, isolated circulating unstimulated DCs from the peripheral blood of healthy HCMV carriers show low levels of expression of viral immediate-early RNA consistent with the induction of reactivation of HCMV in DCs *in vivo* (Reeves and Sinclair, 2013). Our current understanding regarding the myeloid cell differentiation and the role of *in vitro* differentiation and cytokine signaling based on studies on naturally latent HCMV is reviewed in **Figure 1**.

## DEFINING THE DENDRITIC CELL LINEAGE

Beginning in the 1970s, the late Ralph Steinman and colleagues identified and characterized, in a series of studies, a new population of cells with stellate (or dendritic) morphology. These were distinct from other cell types based on a prodigious ability to activate T-cell responses and a biochemical signature that was distinct from that of macrophages (Steinman and Cohn, 1973, 1974; Steinman et al., 1974). Until then, it was generally believed that macrophages were the major orchestrators of the adaptive immune response and, indeed for some time after the initial identification of DCs, this view persisted. However, in a series of elegant experiments Steinman et al. (1983) identified a unique cell population that, although representing less than 1% of the total cells in the spleen, was a major regulator of the immune response (Steinman and Witmer, 1978) and was clearly important for controlling self and non-self recognition – thus illustrating the existence of a cell type that had been postulated by Erhlich some 40 years earlier. Subsequently, it was shown that these cells were resident in a number of tissues known to be at the interface of host pathogen responses as well as highly enriched in lymph nodes of individuals following exposure to pathogens and were eventually termed classical or conventional DCs (cDCs) in order to differentiate them from the subsequently discovered plasmacytoid DCs (reviewed in Banchereau et al., 2000; Merad et al., 2013).

A key aspect in defining the phenotype of DCs is determining their differences from macrophages. Both cell types can arise from monocyte precursors and often share anatomical location. The “text book” view is that macrophages are highly phagocytic and are key players in the innate response, whereas DCs interface with the adaptive response via endocytosis and subsequent processing and presentation of antigens. However, CD11c^-^ macrophages have been shown to activate T-cell responses (Pozzi et al., 2005). Conversely, the well-described, macrophage-induced tissue damage via the induction of cell death pathways, has been suggested for DCs also (Serbina et al., 2003; Stary et al., 2007). Currently, differentiation of the two cell types has largely been based on roles in the innate and adaptive immune response and the expression of cell surface markers. Again, however, the expression of CD68 – a classical macrophage marker – has been detected on myeloid DCs (DC-SIGN-positive cells) resident in human kidney (Segerer et al., 2008). It is interesting to note that CD68 was also detected concomitantly with CD83 on allogenically stimulated monocyte reactivating HCMV (Soderberg-Naucler et al., 1997) suggesting the presence of a heterogeneous phenotype under certain conditions. Given these caveats, it is perhaps not unsurprising that the macrophage/DC system shows a high degree of plasticity between the cell types and may be better regarded as a spectrum of different cell type (Hume et al., 2002). This plasticity would be consistent with both these cell types being members of a larger mononuclear family which share a degree of overlapping functions and ontogeny (Hume et al., 2002).

Despite these caveats, a number of studies have gone on to show that the original cDCs indentified by Steinman and Cohn (1973) represented a unique cell type with distinct biochemical and immunological properties. The DC, existing in an immature state in peripheral blood truly represented the sentinel of the immune response (Banchereau and Steinman, 1998). Since then, the study of DCs has increased exponentially as researchers seek to understand the interaction of DCs with other immune cells in both infection and auto-immunity, as well as trying to harness these cells as potential therapeutic agents against a number of infectious and non-infectious diseases. The very low frequency of DCs in peripheral blood has led investigators to study methods to generate DCs *in vitro* from, predominantly, myeloid precursors and this has driven a substantial expansion of the investigation of these unique cells (Sallusto and Lanzavecchia, 1994; Caux et al., 1997; Strobl et al., 1997a,b). Importantly, a number of functions of these *in vitro* derived DCs have been mapped onto their putative *in vivo* counterparts, based on similar expression of cell surface markers, as well as, more recently, by transcriptional profiling (Merad et al., 2013). Furthermore, augmentation with murine studies has begun to lead to a consensus on their functions with a concomitant discord on the ontogeny of the different DC populations, which is discussed below (Geissmann et al., 2010; Schulz et al., 2012; Merad et al., 2013).

### CLASSICAL DCs

Classical DCs are characterized by an incredibly short half-life (3–5 days) and are replenished from bone marrow precursors in Flt-3L-dependent manner (Liu et al., 2007; Waskow et al., 2008). In general, immature cDCs are highly endocytic and detect pathogens in the periphery. Upon detection of a pathogen, and thus antigen encounter, cDCs efficiently capture, process, and present antigens in the context of MHC molecules (Merad et al., 2013). This maturation is marked by a concomitant up-regulation of a number of T-cell co-stimulatory molecules and migration to lymphoid tissue and, ultimately, presentation of antigen to T cells. This whole process, referred to as the “Langerhans paradigm,” provides the basis of DC activity and function (Banchereau et al., 2000; Merad et al., 2013). Much of our understanding of cDCs comes from studies of the murine system. In the simplest terms, cDCs are divided into two subsets of cells that have differing capacities to present endogenous and exogenous antigens. In the murine system, CD103/CD8a DCs are characterized by the efficient cross-presentation of exogenous antigens via MHC class I to CD8 T cells (Belz et al., 2005; Lundie et al., 2008). In contrast, the CD11b/CD4 cDC subset is a comparatively poor cross-presenter and, instead, is mainly responsible for MHC class II restricted presentation (Dudziak et al., 2007). Mapping these functions onto human counterparts, which is really the key aspect of such studies, has suggested that the CD141 myeloid DC subset are similarly capable of cross-presentation, whereas the CD1c myeloid subset in humans bears the functional hallmarks of the CD11b population of murine DCs (Robbins et al., 2008; Bachem et al., 2010; Crozat et al., 2010). Indeed, it is these human populations of myeloid DCs (the CD1c and CD141 populations) that expressed HCMV immediate-early transcripts *ex vivo* when isolated from the peripheral blood of healthy seropositive individuals (Reeves and Sinclair, 2013).

### MONOCYTES AND MONOCYTE-DERIVED DCs (MoDCs)

Monocytes are key effectors in the immune system retaining the capacity to differentiate into both macrophage and DCs. Again studies in the murine system have suggested the presence of two types of monocytic cell – inflammatory and patrolling monocytes (Geissmann et al., 2008). Although functions have not been directly mapped to human counterparts, it is hypothesized that, in humans, the CD14 low/CD16^+^ and the CD14 high populations represent the patrolling and inflammatory populations identified in mice, respectively (Geissmann et al., 2008). Indeed, recent work in the murine CMV (MCMV) model has postulated that patrolling monocytes are an important vehicle for CMV dissemination – potentially due to their immune-privileged phenotype upon differentiation (Daley-Bauer et al., 2014). Furthermore, it was also hypothesized that these patrolling monocytes may be an important site of MCMV latency but this was not formally addressed in that study. Furthermore, it was in contrast to a previous study that suggested MCMV latency was established in the lung independent of patrolling monocyte function (Marquardt et al., 2011). These differences aside (likely due to differences in experimental procedure, virus strain or route of inoculation for example), a direct analysis of CD16+ and CD16- monocyte populations for the carriage of HCMV genomes in healthy individuals would be extremely informative and could provide pointers to help define the exact population of myeloid cells that carry latent HCMV *in vivo*, which is far from clear. For instance, latency in a sub-population of peripheral monocytes may explain the relatively low frequency of genome-positive cells in healthy individuals (Slobedman and Mocarski, 1999). However, that said, studies from a number of groups have postulated that, at least for mice, patrolling monocytes are derived from inflammatory monocyte precursors (Sunderkötter et al., 2004; Varol et al., 2007; Yona et al., 2013). If this linear route of lineage commitment under steady-state conditions was also the same in humans, it might be predicted that both types of monocytes may be sites of HCMV latency. Nevertheless, very different outcomes of HCMV reactivation could still occur in these different monocyte lineages. For instance, it may be possible that both patrolling and inflammatory monocytes carry latent HCMV but, in normal healthy individuals, reactivation in DCs derived from inflammatory monocytes may be effectively controlled by the immune system and, in contrast, the immune-privileged nature of patrolling monocytes may make these cells important for persistence and transmission. In immune-suppressed individuals, whilst a similar pattern of reactivation may be seen in the absence of a controlling T-cell response, the differentiating inflammatory monocytes would be less likely to be eliminated and may be the source of disease seen in these individuals.

Many studies of the interaction of HCMV with DCs have relied extensively on the generation of DCs from CD14+ (thus predominantly inflammatory monocytes). In a landmark paper, Sallusto and Lanzavecchia (1994) described the generation of DCs from monocytes using interleukin-4 and granulocyte/macrophage-colony stimulating factor (GM-CSF) stimulation which bore many similarities with studies by the Steinman group to derive DCs *in vitro* from peripheral blood progenitors (Romani et al., 1994). This protocol has become the accepted way to generate such MoDCs *in vitro* and results in the generation of highly immune reactive terminally differentiated myeloid DCs and provided strong *in vitro* evidence that monocytes were a precursor of DCs. Although no definitive mapping onto *in vivo* DCs has been achieved thus far, phenotyping studies suggest that MoDCs exhibit an interstitial/dermal DC phenotype rather than a Langerhans (see below) phenotype (Grassi et al., 1998; Duperrier et al., 2000). What is clear is that these *in-vitro*-generated DCs are potent stimulators of a naive T-cell response and have been used extensively to understand host/pathogen interactions *in vitro*.

However, despite these *in vitro* observations, the contribution monocytes made to the DC pool *in vivo* were far from clear. After all, mainly based on studies performed in mice, it was accepted that there was a circulating DC precursor that appeared independently in the blood (Liu et al., 2009). However, these studies *in vitro* argued that monocytes should be DC progenitors *in vivo* and, ultimately, it was shown that microbial stimulation of monocytes in mice promoted the recruitment of DC-SIGN-positive DCs (a marker of MoDCs) to lymph nodes and were distinct from other DC subsets present (Cheong et al., 2010). Indeed, a challenge with a high dose of lipopolysaccharide (LPS) triggered a profound mobilization of the monocyte pool to an effector DC phenotype (Cheong et al., 2010). This response was rapid and strongly supported the hypothesis that monocytes were important for generating DCs *in vivo*.

### LANGERHANS DCs

Langerhans DCs (LDCs) were first described by the German physician Paul Langerhans in 1868 who believed them to be neuronal in origin due to their dendrite-like projections. These cells are resident in the epidermis in an immature state and represent an important first-line response to a number of pathogens as well as a potentially important cell type in Graft versus Host Disease (GvHD) and transplant rejection (Merad et al., 2004, 2008), although this view is still debated (Li et al., 2010). They represent an extremely long-lived population of cells that have the capacity for self-renewal in the periphery (Czernielewski and Demarchez, 1987). They can be generated *in vitro* from both CD34+ and CD14+ precursors and are characterized by the expression of Birbeck granules, Langerin, and E-Cadherin (Strobl et al., 1997a; Geissmann et al., 1998). The cytokine TGF-b appears important for the generation of LDCs *in vitro* and *in vivo* (Borkowski et al., 1996; Strobl et al., 1997b) although it is becoming increasingly clear they have a contentious ontogeny (Merad et al., 2002; Ginhoux et al., 2006; Chorro and Geissmann, 2010; Schulz et al., 2012). Although they can be generated *in vitro* from myeloid precursors, whether this is the case *in vivo* clearly remains a point of debate. Studies in murine models have suggested that LDCs are seeded predominantly from the yolk sac (Schulz et al., 2012) during very early embryogenesis and that the adult LDCs are subsequently replenished by the localized pool of immature LDCs in the periphery rather than a myeloid progenitor cell (Merad et al., 2002). Furthermore, the precise contribution of the yolk sac to the Langerhans cells resident in adult tissue is also debated – while the yolk sac clearly contributes to the LDC pool in the periphery it has been postulated that second wave of LDCs are seeded primary in later developmental stages from fetal liver monocytes (Hoeffel et al., 2012). Consistent with monocytes having the potential to seed LDCs, studies have shown that LCs can be re-populated from circulating monocytes in the peripheral blood (Ginhoux et al., 2006) and thus the precise ontogeny of the LDC lineage is still debated. From these many studies, it is becoming clear that, as techniques for separating cell populations become more sensitive, an ever increasing number of subsets of peripheral DCs are becoming discernible and their differences may be due, in part, to the criteria different investigators are using to define them. Furthermore, caution may also be required when translating studies in the murine model directly onto the human system (Mestas and Hughes, 2004; Seok et al., 2013).

However, the concept of LDCs exhibiting longevity in the periphery may be important in the context of HCMV biology. Studies have shown that, *in vitro*, immature LDCs are not permissive for lytic infection and thus, potentially, HCMV could establish a latent infection in these cells. As such, whether there is a pool of LDCs resident in the periphery which is HCMV genome positive is an important question. Furthermore, if circulating monocytes can re-populate this LDC pool in the periphery in response to inflammation (Ginhoux et al., 2006), this, again, could lend itself to the establishment of a site of HCMV latency in the periphery.

### PLASMACYTOID DCs

Plasmacytoid DCs (pDCs) represent a distinct lineage considered to be of lymphoid origin (Reizis et al., 2011). A key feature of pDCs is the ability to secrete large quantities of type 1 interferons in response to pathogens and, morphologically, they show little resemblance to their myeloid counterparts, exhibiting a spherical morphology consistent with their lymphoid origin (Grouard et al., 1997; Cella et al., 1999). Indeed, they also share little commonalities with cDCs. In general, they are not phagocytic and, as such, represent poor presenters of exogenous antigen (Gehrie et al., 2010) – although, interestingly, their generation is also dependent on Flt-3L (Maraskovsky et al., 2000). Consistent with their dissimilarity to classical myeloid DCs, there is no evidence to suggest they are important sites of HCMV latency and reactivation and, indeed, are non-permissive for HCMV lytic infection (Kvale et al., 2006).

## *IN VITRO* GENERATION OF MYELOID DC SUBTYPES

DCs *in vivo* are constantly re-populated from the haematopoietic stem cell compartment with fate-mapping studies suggesting that the majority of DCs, including pDCs of lymphoid origin, arise from the common myeloid progenitor (CMP, Manz et al., 2001). The macrophage-DC progenitor (MDCP) that arises from the CMP is the source of pDCs, cDCs, monocytes, and macrophages but, interestingly in the context of HCMV biology, not the granulocyte or other myeloid populations (Auffray et al., 2009). Further progenitor cell types, including the common DC progenitor, have been identified in mice and it is likely these observations map onto human haematopoiesis. As such, the generation of terminally differentiated myeloid cell types from multiple progenitors has been demonstrated.

In the context of the analysis of HCMV biology, two cell types – the CD34+ progenitors and CD14+ monocytes – have been used extensively. As discussed above, CD14+ monocytes can be differentiated into DCs using IL-4 and GM-CSF (Sallusto and Lanzavecchia, 1994). These DCs have been proposed to resemble dermal DCs. The addition of transforming growth factor-beta (TGF-b) to these cultures promotes a Langerhans-like phenotype (Geissmann et al., 1998), consistent with the known role of TGF-b for LDC formation *in vivo* (Borkowski et al., 1996). In both instances, differentiation is marked by limited cellular proliferation and cellular identity is determined by the expression levels of a panel of markers. Indeed, to date, a single definitive marker of DC has not been identified.

Myeloid DCs (including both interstitial and Langerhans DCs) can also be generated from bone marrow CD34+ progenitors using GM-CSF and tumor necrosis factor-alpha (TNF-α) (Caux et al., 1997). These cultures are associated with significant expansion, particularly following the addition of stem cell factor which acts synergistically with GM-CSF and TNF-α to drive proliferation. Furthermore, as observed with monocyte-derived Langerhans cells (Geissmann et al., 1998), the addition of TGF-b has a profound effect on phenotype promoting the formation of predominantly Langerhans cultures (Strobl et al., 1997a,b). The addition of Flt-3L to the mix was subsequently shown to be important for successful expansion of CD34+ cells isolated from the peripheral blood of G-CSF mobilized donors and, to date, has been a standard technique for generating LCs for studies of HCMV biology.

## VIRAL SUBVERSION OF DC FUNCTION

### LYTIC INFECTION OF DCs

Much of our understanding of the effect of HCMV on DCs has been derived from studies on their lytic infection and has been reviewed recently (Hertel, 2014) and thus the focus of this review is on the interaction of latent HCMV with DCs. Importantly, studies on HCMV differentiation-dependent lytic infection of cell lines have illustrated that there is a direct correlation between permissiveness for lytic infection and virus reactivation and that these observations are re-capitulated in MoDCs (Gönczöl et al., 1985; Weinshenker et al., 1988; Lathey and Spector, 1991; Riegler et al., 2000; Hertel et al., 2003; Reeves et al., 2005b). The evidence that DCs were permissive for HCMV infection was provided by the Jahn laboratory using the highly endothelial-tropic virus, TB40/e, to productively infect MoDCs (Riegler et al., 2000). Infection of both immature and mature DCs was observed – although, in their hands, the infection of immature MoDCs appeared to be more efficient. These observations were then complemented by studies from the Mocarski laboratory who showed that LDCs were also susceptible to HCMV infection but, unlike MoDCs, only after CD40L-induced maturation (Hertel et al., 2003). Subsequent studies have shown that DCs directly isolated from peripheral blood are also permissive for infection (Kvale et al., 2006; Reeves and Sinclair, 2013).

What became clear from such data was that HCMV infection of DCs resulted in a severe down-regulation of cell surface molecules which impacted on the normal function of DCs as APCs (Riegler et al., 2000; Grigoleit et al., 2002; Moutaftsi et al., 2002, 2004; Hertel et al., 2003; Lee et al., 2006, 2011; Huang et al., 2012). Although viral genes encoding immune evasion functions are expressed in all cell types, the sheer abundance of immune-evasins expressed by the virus suggests the evolution of an intimate relationship between HCMV and key immune effector cells – e.g., the DC lineage. However, it is worth remembering that, despite all these immune evasion functions encoded by the virus, the immune system in healthy individuals still appears to adequately control the virus and is able to routinely prevent clinical pathology. Consequently, the functional consequences of this abundance of viral immune evasion strategies are far from clear during primary infection or reactivation *in vivo*. Work on the rhesus model has illustrated that the US2-11 genes are non-essential for primary infection of CMV-negative animals (Hansen et al., 2010), whereas the expression of these genes was crucial for infection in the presence of pre-existing immune response to CMV (Hansen et al., 2010). It remains to be determined whether the expression of US2-11 may also be important during reactivation from latency or is solely required for re-infection of hosts with pre-existing immunity to HCMV. Indeed, the immediate-early kinetics of some of these transcripts arising from the US2-11 region (e.g., US3) during primary lytic infection (Tenney and Colberg-Poley, 1991) could argue for a potential role in the early stages of HCMV reactivation, if expression of these RNAs during reactivation exhibited the same expression kinetics as during primary infection. The inhibition of antigen presentation, particularly of the immune-dominant IE72 peptides, may provide a window of opportunity for the virus to reactivate before the immune system regains control.

## HCMV REACTIVATION IN DCs

As stated above, the permissiveness for both lytic infection and HCMV reactivation in the myeloid lineage is also closely linked with the differentiation status of the cell. Given that the initiation of a lytic infection requires the prodigious activities of both the IE72 and IE86 proteins, whose expression is driven by the major immediate-early promoter (MIEP), it has been hypothesized that differentiation-dependent regulation of the MIEP underpins both permissiveness for infection of a myeloid cell type as well as reactivation from latency (Sinclair and Sissons, 1996).

First, in the case of infection of a permissive cell type, there is a pre-requisite for the virus to overcome intrinsic blocks to viral IE gene expression and, in most differentiated cell types (including DCs), the virus achieves this allowing productive infection to proceed. In contrast, in a non-permissive cell, the virus cannot overcome these intrinsic blocks and continued suppression of the MIEP allows the establishment of a quiescent infection that can ultimately result in viral latency in, for instance, a monocyte or CD34+ cell. Alternatively, during the scenario of reactivation, latent/quiescent genomes transit from a nuclear environment that does not support MIEP activity to one that does. This reactivation of IE gene expression – the pivotal first step toward complete reactivation of infectious virus – must occur in an absence of other viral factors or virally induced signaling which likely make a profound contribution to MIEP activity upon the de novo infection of cells permissive for HCMV.

One simple hypothesis which fits these data is that cellular differentiation increases the availability of cellular transcription factors that promote MIEP activity and/or decreases the levels of cellular factors which suppress MIEP activity – but the evidence for this in primary cells is limited. That said, we do know that the transfected MIEP is inherently less active in undifferentiated, compared to differentiated, myeloid cells (Sinclair et al., 1992)- which would be consistent with the differentiation dependence of MIEP activity resulting from positive/negative transcription factor binding and availability. However, other data suggest that this linear hypothesis may represent a too simplistic relationship and a number of other requirements need to be met to facilitate virus reactivation at the level of the MIEP.

Our first studies on reactivation of HCMV from LCs derived from CD34+ of healthy seropositive donors were entirely consistent with their concomitant permissiveness for lytic infection; there was an absolute requirement for maturation (with LPS or CD40L) after differentiation for HCMV reactivation (Reeves et al., 2005a,b) and experimental lytic infection to occur (Hertel et al., 2003). Similarly, more recent work using monocyte-derived LCs (MoLCs) also shows that maturation of these cells is required for both HCMV reactivation and their permissiveness for lytic infection (Huang et al., 2012).

The story regarding reactivation from MoDCs *in vitro* was less clear. In our initial studies, the detection of HCMV reactivation from naturally latent individuals required LPS-induced maturation of the DCs (Reeves et al., 2005b). Thus, although immature DCs were permissive for HCMV lytic infection (Riegler et al., 2000) they did not appear to support detectable reactivation. However, what became evident in subsequent studies was that immature DCs could support HCMV reactivation if stimulated with the inflammatory cytokine interleukin-6 – circumventing the need for maturation (Reeves and Compton, 2011). Failure to reactivate in these immature DCs was not due to an intrinsic block to reactivation but more to a lack of a robust signal to drive the expression of the IE genes critical for reactivation to occur.

Indeed, some of the very first observations of HCMV reactivation in the DC lineage utilized a highly pro-inflammatory cytokine environment to support reactivation (Soderberg-Naucler et al., 1997; Hahn et al., 1998). DCs themselves are prodigious producers of inflammatory cytokines, particularly upon stimulation with bacterial products such as LPS which probably drives the reactivation observed *in vitro* using these cell types. Furthermore, models of HCMV reactivation have usually relied on co-culture with fibroblasts (Hahn et al., 1998; Goodrum et al., 2002; Reeves et al., 2005a) which are similarly efficient cytokine producers – and this includes interleukin-6 (Sundararaj et al., 2009). Interestingly, treatment of long-term culture of non-adherent experimentally latent monocytes in media supplemented with interleukin-6 (IL-6) has been shown to induce HCMV reactivation (Hargett and Shenk, 2010) and, similarly, the LPS-mediated reactivation of HCMV from experimentally latent immature DCs has been shown to be dependent on IL-6 (Reeves and Compton, 2011). Perhaps not unsurprisingly, then, the addition of IL-6 directly to immature DCs is known to circumvent the need to induce maturation and, reciprocally, the ablation of IL-6 signaling in LPS-treated immature DCs is sufficient to impede virus reactivation (Reeves and Compton, 2011).

In order to understand the mechanisms controlling HCMV reactivation it becomes important to define what is understood by the repression of the MIEP during latency. For instance, is the MIEP completely silenced or is the MIEP just significantly repressed to prevent the initiation of the lytic replication cycle? As such, does inflammation just enhance the activity of the MIEP rather than trigger reactivation? Indeed, the evidence of transcription initiation from “repressed” cellular genes in stem cells (Guenther et al., 2007) suggests that sporadic initiation of RNA Pol II activity at the MIEP may also be expected. Accordingly, the existence of a virally encoded miRNA species that targets the major IE transcript (Grey et al., 2007) would be consistent with a requirement to regulate IE expression post-transcriptionally, at least during some stage of the viral lifecycle. Since it is unlikely that such an miRNA would counter the super-abundance of the IE transcripts during lytic infection, it is possible that such an RNAi-based mechanism could be important during HCMV latency (Grey et al., 2005, 2007; Murphy et al., 2008). Indeed, expression of the UL122 and UL123 transcripts from the MIE region is unlikely to have a functional impact if they remain untranslated.

There is good evidence that cellular factors play an important role in regulating the MIEP during latency and reactivation in a differentiation dependent manner (reviewed in Reeves and Sinclair, 2008). Furthermore, more recent data suggest that the establishment and maintenance of HCMV latency may be dictated by the activity of viral factors. For instance, the exclusion of pp71 from the nuclei in CD34+ cells during the initial stages of infection (Saffert et al., 2010) combined with the recruitment of the polycomb repressor complex by the viral lncRNA4.9 transcript to the MIEP (Rossetto et al., 2013) may contribute to establishment and maintenance of the latent state. Our own work in primary DCs (Kew et al., 2014), along with studies in cell lines which show a differentiation-dependency for infection, points toward an important role for the CREB response elements within the MIEP for HCMV reactivation (Keller et al., 2007; Yuan et al., 2009). However, it is of note that around 4000 genes exhibit CREB occupancy in the human genome (Zhang et al., 2005). Unsurprisingly, agonists of the cAMP pathway, whilst promoting CREB phosphorylation, did not necessarily trigger gene expression from all these loci (Zhang et al., 2005). Critically, the cellular co-factors available to interact with CREB are likely to dictate specific responses. Consequently, it is thus likely that a similar requirement for co-factor availability will also drive responsiveness of the MIEP to such signals in multiple cell types. Thus, integrating the role of inflammation, cellular signaling, and cellular differentiation will provide the basis for delineating the mechanisms that govern MIEP regulation during HCMV latency and reactivation.

## DOES HCMV REACTIVATE IN AN IMMUNE-DEFICIENT ENVIRONMENT?

The observation that HCMV maintains latency and reactivates in the archetypal control cell of the adaptive immune response may, at first glance, seem like a flawed strategy – virus reactivation occurs in cells which, themselves, mediate the activation of a potent arm of the anti-pathogen immune response and which could be argued to be able to promote highly efficient control of HCMV infection. Indeed, the known memory inflation of T cells that recognize HCMV in healthy individuals does suggest regular immune priming events which could be due to regular low-level reactivation which, for the most part, is asymptomatic.

Alternatively, reactivation in terminally differentiated cells of the myeloid lineage could be considered analogous to the Trojan horse model whereby the virus is actually carried and reactivates in the optimum cell type for the virus to control host immune responses, thereby permitting efficient viral persistence. Clearly, the surfeit of immune evasion molecules encoded by the virus would lend itself enormously to this type of “hijacking” of normal host immune control mechanisms. In essence, by reactivating in DCs the virus is positioned to control the bioactivity of a cell type which is crucial to the control of viral infections.

Whilst reactivation of viral lytic infection in terminally differentiated myeloid cells will almost certainly result in a transcription program essentially akin to an experimental lytic infection this, to our knowledge, has not been tested directly. In contrast, in latently infected cells there is, as yet, no compelling evidence to suggest that such functions are expressed. One virally encoded gene with potent immune modulatory effects, however, is known to be expressed during latent infection, i.e., viral interleukin-10 (vIL10; Jenkins et al., 2008b).

### INTERLEUKIN-10

Interleukin-10 (IL-10) is major cellular cytokine with well-documented, immune-suppressive properties (Opal and Huber, 2000). In normal individuals, IL-10 expression likely represents the counter-effect to excessive inflammation providing a negative feedback to maintain homeostasis in the immune system rather than promoting immune-suppression. However, evidence suggests that HCMV harnesses IL-10, both cellular and via virally encoded IL-10 homologues, to drive an immune-dampening phenotype that could enhance both lytic and latent infection (Slobedman et al., 2010; Mason et al., 2012).

Cellular IL-10 (cIL-10) homologues encoded by HCMV (vIL-10) are known to be expressed in both lytic and latent infection (Jenkins et al., 2004, 2008a; Cheung et al., 2006). Reviewed extensively elsewhere, a number of studies using the rhesus CMV model have illustrated that vIL-10 proteins are important for dissemination of virus upon primary infection (Chang and Barry, 2010) and, combined with studies of HCMV *in vitro*, that this could be due to a functional impact of vIL-10 on MHC class II expression along with an immune-dampening effect on T-cell responses to HCMV infected cells (Spencer et al., 2002; Chang et al., 2004; Raftery et al., 2004; Cheung et al., 2009). Furthermore, there is evidence that vIL10 expressed during latency may promote a non-classical differentiated myeloid phenotype (Avdic et al., 2011, 2013). For instance, the expression of the latent form of vIL10 in myeloid progenitors has a dramatic impact on cellular differentiation - in infected monocytes, vIL10 drives monocytes to an M2c macrophage phenotype which is known to have diminished immune responses (Avdic et al., 2013). Furthermore, expression of vIL-10 in granulocyte-macrophage progenitors abrogates their differentiation to a classical DC phenotype (Avdic et al., 2011). Thus, both strategies would contribute to successful persistence of the virus in the host by preventing normal recognition of a latently infected cell by the immune response. Furthermore, in the light of recent work that has studied the role of patrolling monocytes in the dissemination of MCMV (Daley-Bauer et al., 2014) vIL-10 may play other roles. For instance, patrolling monocytes can generate anti-inflammatory signature in response to pathogens which is exemplified by IL-10 production. Therefore, it is possible that the immune-privileged phenotype associated with these monocytes could also involve IL-10 and in the context of CMV infection, enhance viral dissemination.

cIL-10 also exerts a number of effects on APCs and on the T cells that interact with them. For instance, addition of cIL-10 to APCs promotes significant down-regulation of MHC class II surface expression which impacts on CD4 T-cell responses (Koppelman et al., 1997). Furthermore, cIL-10 also imparts a functional effect on T cells – driving the recruitment of immune-suppressive cells on an cIL-10 gradient and acting to suppress the function of classical effector CD 4 T cells (Groux et al., 1996). Indeed, it is hypothesized that the induction of cIL-10 (with TGF-b) by latently HCMV-infected CD34+ cells contributes to the generation of an immune-suppressive environment in the proximity of latently infected cells, thus protecting them from elimination by the host immune response (Mason et al., 2012).

### US GENES AND VIRAL miRNAs

Human cytomegalovirus encodes a number of gene products that are important for regulating both MHC class I- and class II-mediated antigen presentation. These proteins (expressed from genes encoded by the US2-11 region of HCMV) target multiple aspects of the antigen presentation pathway in an attempt to render the infected cell invisible to the immune response (Powers et al., 2008). Expressed throughout lytic infection, these viral gene products target MHC surface expression, antigen processing, and loading as well as the delivery of peptide-loaded MHC molecules to the cell surface. The evolution of multiple immune evasion genes suggests a strong selective pressure on the virus to evolve robust counter-measures to a sophisticated host immune response. As expected, these genes are non-essential *in vitro* and studies with rhesus CMV (RhCMV) have also suggested they are dispensable for primary infection also (Hansen et al., 2010). Instead, the US gene products (particularly, 2, 3, 6, and 11) were required for super-infection of rhesus macaques with RhCMV (Hansen et al., 2010). Thus, it is hypothesized that the US 2-11 genes are required for re-infection of a host with a pre-existing immunity to CMV. The reason for this is unclear but, potentially, the capacity of the virus to super-infect individuals with pre-existing anti-viral immunity could increase the potential for genetic recombination between multiple strains of HCMV which could impact on virus persistence *in vivo*. Clearly, the expression of these potent immune-evasins in DCs reactivating HCMV likely plays an important role in efficiency of virus reactivation to allow horizontal and vertical transmission as well as, possibly, the maintenance of the latent reservoir.

The expression of such an effective repertoire of immune evasion molecules during lytic infection begs the question as to why HCMV also encodes at least one miRNA which is involved in de-regulating antigen processing and presentation. This virally encoded miRNA, miRNA US4-1, has been shown to target ERAP – a key cellular protein for the processing of antigens for MHC presentation (Kim et al., 2011). However, why additional mechanisms involving miRNAs might be required to target antigen presentation during lytic infection when such cellular functions are already extensively targeted by key viral immune evasion proteins are intriguing. It is possible that miRUS4-1 has additional functions or that that it provides a “belt and braces” approach to ensure efficient regulation of antigen presentation by the virus. Similarly, whether the real function lies during latency (where many viral proteins are not expressed) or in cell types other than fibroblasts (where viral gene expression may be less efficient) remains to be determined.

## INFLAMMATION AND HCMV

The contribution of the cellular environment to HCMV reactivation has been studied intensely using models of myeloid cell differentiation. An important aspect that defines the cellular environment is the action of the extra-cellular milieu. Indeed, inflammation and HCMV viraemia and disease in a number of patient populations are closely linked (Humar et al., 1999; Blankenberg et al., 2001). Consistent with a role for inflammation, a number of studies of HCMV reactivation have suggested that key inflammatory cytokines such as TNF-α, IL-6, and interferon-y could be important factors for reactivation (Humar et al., 1999). Hypothetically, HCMV disease may be linked to the inflammation associated with allograft rejection and GvHD. HCMV reactivation and disease have been suggested to be the risk factors in sepsis patients (Heininger et al., 2001) and indeed LPS is a prodigious stimulator of cytokine production in multiple cell types. In contrast, the use of anti-inflammatory treatments (which may be predicted to reduce HCMV reactivation through diminished inflammation), such as TNF antagonists like ENTERCERPT, to treat auto-immune diseases, has been associated with increased herpes virus reactivation (Kim and Solomon, 2010). Consequently, attempts to dampen the auto-immune response with such anti-inflammatory drugs are likely to impact on the normal regulation of HCMV reactivation also. Essentially, reducing inflammation may diminish the level of HCMV reactivation observed at a molecular level. However, reduced immune activity associated with anti-inflammatory treatments may have a negative impact on the level of immune control normally exerted on HCMV in healthy individuals.

One *in vitro* model for HCMV reactivation from monocytes specifically uses IL-6-induced signaling via the ERK-MAPK pathway (Reeves and Compton, 2011). Clearly, many cytokines could potentially activate this signaling pathway – so an obvious question is whether IL-6, itself, is important for HCMV reactivation *in vivo*. Unfortunately, the limited clinical data are not equivocal. Aggressive HCMV disease is known to be associated with elevated IL-6 serum levels (Humar et al., 1999; Blankenberg et al., 2001). However, such studies were not designed to distinguish between a potential role for inflammation in virus reactivation and the clinical outcomes of induction of inflammation during viraemia.

It is known that systemic IL-6 production is important for overcoming immune-suppression, elicited by T regulatory cells, by driving a T effector cell function (Wan et al., 2007) – an event that has been speculated to be particularly important in graft rejection (Liang et al., 2007). Consequently, why the induction of HCMV reactivation in response to IL-6 has evolved is unclear – as it would result in reactivation in the presence of a strong anti-viral immune response. Intriguingly though, IL-6 has unique effects on DCs *in vitro*. In essence, treatment of DCs with IL-6 results in the generation of a DC cell type that remains functionally immature compared with LPS or CD40L activated DCs (Hegde et al., 2004). This effect of IL-6 on DCs potentially generates a cell type that supports HCMV reactivation but, at the same time, is not fully capable of acting most effectively as an antigen presenting cell. Taken together, the fact that HCMV encoded vIL10 is also immune-suppressive (Spencer et al., 2002; Jenkins et al., 2008b) and latent infection of CD34+ cells results in induction of immune-suppressive cIL-10 (Mason et al., 2012) suggests that HCMV benefits from manipulating the DC cell to an intermediate phenotype which is functionally just short of mature classical DCs or macrophages (Avdic et al., 2013).

Whether IL-6 is a “master cytokine” *in vivo* for HCMV reactivation is unclear. Human cytomegalovirus reactivation from monocytes, as a result of allogeneic T-cell stimulation (Soderberg-Naucler et al., 1997), appeared to result from the extensive induction of inflammatory cytokines, one of which was IL-6 (Soderberg-Naucler et al., 2001). However, high levels of many other cytokines such as TNF-α and interferon gamma were also detected – and antibody neutralization of both of these abrogated virus reactivation (Soderberg-Naucler et al., 2001). Indeed, it has been hypothesized that the coincidence of HCMV reactivation with GvHD (which is also driven by allogeneic T-cell activation) is mimicked in this system and that, *in vivo*, HCMV might respond to the excessive inflammation associated with GvHD. Of course, viral reactivation and re-entry into the lytic cycle will likely result in inflammation, in itself, and thus a destructive positive feedback loop could exacerbate GvHD in these CMV-infected individuals. Ultimately, the “litmus test” would be to determine whether using neutralizing antibodies against IL-6 reduces HCMV reactivation *in vivo*.

TNF-α has also long term been proposed to be important for CMV reactivation *in vitro* and *in vivo* (Prösch et al., 1999, 2002; Hummel and Abecassis, 2002; Cook, 2007) and, consistent with this, it is well known that the viral MIEP responds to NF-kB which is a major downstream effector molecule of the TNF-α signaling cascade (Cherrington and Mocarski, 1989; Sambucetti et al., 1989). Indeed, TNF-α has been used to reactivate HCMV in a number of cell line models as well as reactivate MCMV in murine models of virus reactivation (Hummel and Abecassis, 2002; Prösch et al., 2002; O’Connor and Murphy, 2012). As such, TNF-α antagonists have been proposed as potential measures to control HCMV reactivation *in vivo* but, as of yet, no unequivocal evidence has been presented to confirm this. Indirect support for a role for TNF-α in HCMV pathogenesis is suggested by the known modulation of tumor necrosis factor receptor one (TNFRI) expression on the surface of infected cells by HCMV infection. For instance, both clinical and laboratory isolates of HCMV down-regulate TNFRI early during lytic infection (Baillie et al., 2003; Le et al., 2011; Montag et al., 2011). However, recently, the UL138 gene product encoded by HCMV in its ULb’ region has also been shown to up-regulate TNFR1 cell surface expression (Le et al., 2011; Montag et al., 2011) – this, perhaps, points to a need for the virus to modulate this important signaling cascade differentially during the course of lytic infection. Importantly, UL138 is also known to be expressed during latent infection (Goodrum et al., 2007) suggesting that HCMV may render cells more responsive to TNFRI and, thus, potentially prime cells for reactivation. However, formal proof of this in the context of latent viral infection remains to be shown.

Indeed, when we begin to consider HCMV reactivation in the context of myeloid cell differentiation and inflammation, a number of important questions arise. The differentiation-dependent model of latency predicts that, in early progenitor cells, the latent HCMV genome is relatively unresponsive to stimuli that would normally be expected to activate the MIEP. Clearly, cellular differentiation, *per se*, triggers HCMV reactivation in a number of models – an event that is exacerbated by inflammation. However, *in vitro*, cytokine stimulation forces the differentiation of myeloid cells down specific lineages which may not fully reflect events *in vivo*. For instance, monocytes isolated from HCMV viraemic patients display a defect in a GM-CSF paracrine signaling which, ultimately, could impair their normal differentiation and immune function (Carlier et al., 2011). Of course, viraemia represents a specific condition but it remains to be seen if HCMV reactivation exerts any effect on the normal differentiation of myeloid cells in healthy individuals. It is revealing that the effects on GM-CSF paracrine signaling were mediated by IL-6 (Carlier et al., 2011) and IL-6 has been shown to trigger higher levels of HCMV reactivation from monocytes in long-term culture (Hargett and Shenk, 2010) and differentiating DCs (Reeves and Compton, 2011; Huang et al., 2012). It is tempting to speculate that HCMV modulates the cellular microenvironment to promote HCMV reactivation which, in healthy individuals, could contribute to persistence of the virus. However, in the context of immune-compromised individuals this becomes a source for uncontrolled virus replication and dissemination resulting in severe morbidity. HCMV clearly expresses a bioactive secretome during latency *in vitro* (Mason et al., 2012) which would be augmented by the activity of virally encoded functions such as vIL-10 (Slobedman et al., 2010). Taken together, these activities may also be responsible for modulating the function of differentiating monocytes and myeloid precursors to a phenotype that is relatively immune-suppressive (Hargett and Shenk, 2010; Avdic et al., 2011, 2013). Thus, while the model predicts that cellular differentiation is important for HCMV reactivation, it is becoming clear that the virus could hijack specific pathways to modulate cellular functions to support reactivation in a cell type that is not a classical DC.

## FINAL COMMENTS

Human cytomegalovirus exhibits a very close relationship with the cells of the myeloid lineage – and particularly macrophages and DCs. DCs likely represent an important cell type for the resolution of primary infection and also for the control of reactivation. The HCMV genome is dominated by gene products that are important for immune-modulation including a significant number of genes that target components of the adaptive immune response. A key role of DCs is to respond to pathogens to elicit an effective immune response via the activation of T lymphocytes. Part of the response of DCs involves significant activation of key inflammatory cytokines and chemokines that contribute to the successful resolution of the infection. Interestingly, the viral MIEP exhibits a number of similarities with the promoters of genes encoding these key inflammatory mediators, suggesting that the viral MIEP behaves as a cryptic inflammatory promoter which responds to the same regulatory inflammatory signals. In itself, this may still prove to be ineffective for the virus in the face of a normal and robust immune response. However, when reactivation is considered in the context of the range of other strategies HCMV employs to diminish the strength of the immune response during latency and reactivation, reactivation in immunologically “impaired” DCs could provide the ideal spring-board for viral reactivation, dissemination and, possibly, transmission. In the setting of, often iatrogenic, host immune-suppression this delicate balance is dramatically tipped toward the virus where it results in the serous morbidity often associated with this opportunistic pathogen. The general belief that myeloid cell differentiation along with concomitant inflammatory signaling is important for triggering HCMV reactivation in the myeloid lineage is supported by a wealth of data However, as we begin to understand the increasingly complex interaction of HCMV with the immune system, it will become fundamentally important to dissect the mechanisms HCMV employs to usurp the normal biology of myeloid cells such as DCs to facilitate viral reactivation and the successful persistence of this opportunistic pathogen.

## Conflict of Interest Statement

The authors declare that the research was conducted in the absence of any commercial or financial relationships that could be construed as a potential conflict of interest.
